# Toward Safer Diagnoses: A SEIPS-Based Narrative Review of Diagnostic Errors

**DOI:** 10.3390/diagnostics16020347

**Published:** 2026-01-21

**Authors:** Carol Yen, John W. Epling, Michelle Rockwell, Monifa Vaughn-Cooke

**Affiliations:** 1 Department of Health Systems and Implementation Science, Virginia Tech School of Medicine, Roanoke, VA 24014, USA; carolyen@vt.edu; 2 Department of Industrial and Systems Engineering, Virginia Polytechnic Institute & State University, Blacksburg, VA 24061, USA; 3 Carilion Clinic and Virginia Tech Carilion School of Medicine, Roanoke, VA 24014, USA; jwepling@carilionclinic.org; 4 Department of Human Nutrition, Foods, and Exercise, Virginia Polytechnic Institute & State University, Blacksburg, VA 24061, USA; mrockwell@carilionclinic.org

**Keywords:** patient safety, socio-behavioral factors, cognitive bias, health literacy, sociotechnical systems, interpersonal relationships, diagnostic decision making, diagnostic communication, health information technology, system-level contributors

## Abstract

Diagnostic errors have been a critical concern in healthcare, leading to substantial financial burdens and serious threats to patient safety. The Improving Diagnosis in Health Care report by the National Academies of Sciences, Engineering, and Medicine (NASEM) defines diagnostic errors, focusing on accuracy, timeliness, and communication, which are influenced by clinical knowledge and the broader healthcare system. This review aims to integrate existing literature on diagnostic error from a systems-based perspective and examine the factors across various domains to present a comprehensive picture of the topic. A narrative literature review was structured upon the Systems Engineering Initiative for Patient Safety (SEIPS) model that focuses on six domains central to the diagnostic process: Diagnostic Team Members, Tasks, Technologies and Tools, Organization, Physical Environment, and External Environment. Studies on contributing factors for diagnostic error in these domains were identified and integrated. The findings reveal that the effectiveness of diagnostics is influenced by complex, interconnected factors spanning all six SEIPS domains. In particular, socio-behavioral factors, such as team communication, cognitive bias, and workload, and environmental pressures, stand out as significant but difficult-to-capture contributors in traditional and commonly used data resources like electronic health records (EHRs), which limits the scope of many studies on diagnostic errors. Factors associated with diagnostic errors are often interconnected across healthcare system stakeholders and organizations. Future research should address both technical and behavioral elements within the diagnostic ecosystem to reduce errors and enhance patient outcomes.

## 1. Introduction

Diagnostic error is a critical and prevalent issue in healthcare, posing major threats to patient safety and national healthcare costs [1,2] and leading to significant harm, including preventable deaths [3]. The unpredictable and evolving nature of disease conditions makes diagnostic safety more challenging to address [4] than other healthcare safety issues, such as medication and surgical errors [1,5]. The complexity of defining and quantifying diagnostic error shows that this area is largely overlooked in the field of patient safety when compared to other quality concerns, including infection, wrong-site surgery, and adverse drug events [4,6,7]. Approximately 5% of patients in U.S. outpatient settings suffer from diagnostic errors [8], and most people experience at least one diagnostic error in their lifetime [9]. Diagnostic errors also create a significant financial burden on the U.S. healthcare system. The National Academy of Medicine estimates that about 25% of annual healthcare spending (ranging from $760 billion to $935 billion) is wasted on unnecessary services and inefficiencies, although the exact contribution of diagnostic errors remains unclear [10]. However, other estimates of the costs associated with misdiagnosis-related harms, ranging from mild to severe, may exceed $100 billion per year [11].

In 2015, the National Academies of Sciences, Engineering, and Medicine (NASEM) published a pioneering report named Improving Diagnosis in Health Care [9]. This report underscores the importance of diagnostic errors and the lack of a clear and precise framework to identify the errors. The committee defined diagnostic errors as the failure to (a) establish an accurate and timely explanation of the patient’s health problem(s) or (b) communicate that explanation to the patient [9]. The definition highlights the importance of accuracy, timeliness, and communication among stakeholders in fostering a more complete understanding of diseases and diagnoses. Diagnostic accuracy refers to the ability to identify the disease correctly and to discriminate between a patient with or without the target health condition [12,13]. Timeliness can be determined by the time interval between symptom onset to the first consultation, referral, and diagnosis of the disease [14,15]. Communication is a crucial component through the whole diagnostic process, bridging the information exchange between each stakeholder [16,17]. Meaningful and effective communication is a goal not just for patients or clinicians but for all participants in the diagnostic journey.

In addition to developing comprehensive definitions for diagnostic error, identifying contributory factors is also a key element in understanding how diagnostic outcomes are generated. Diagnostic error research has primarily focused on capturing disease-related factors [18,19,20,21], including laboratory results, imaging, and treatments, which are easier to extract from medical records. Studies that capture diagnostic error have mainly emphasized timeliness and accuracy, due to the complexity of identifying and documenting communication-related diagnostic errors. Despite the wealth of information contained in medical records, socio-behavioral data are commonly excluded [22,23]. For example, patient characteristics, such as health literacy and disease awareness, affect how individuals can articulate their symptoms and understand treatment instructions, which directly impacts the diagnostic process [24,25]. The quality and completeness of information exchange are shaped by both communication and the relationship between patient and clinician, which directly impacts diagnostic outcomes [26]. Beyond individual interactions, clinician cognitive bias has received significant attention for its direct impact on the decision making process, potentially leading to judgment errors [27]. At the organizational level, challenges such as excessive administrative burdens [28,29], clinician burnout [29,30], and tensions in peer [31] and hierarchical relationships [32] impact diagnostic accuracy and the overall quality of care.

Although many contributors to diagnostic error have been identified in the previous literature, they are typically discussed in isolation rather than as part of an interacting system [25,33]. This fragmentation makes it challenging to understand how clinical, organizational, technological, and environmental factors collectively shape the complete picture of diagnosis [34]. To address this gap, this review synthesizes the literature using a sociotechnical framework adapted from the Systems Engineering Initiative for Patient Safety (SEIPS) model to organize system-level contributors to diagnostic error. By integrating evidence across diverse clinical contexts and methodological approaches, this review aims to identify generalizable patterns and interactions that can guide the development of targeted strategies to improve diagnostic safety.

## 2. Methods

### 2.1. Framework Orientation and SEIPS Adaptation

This review is grounded in the SEIPS model [25], a human-centered sociotechnical framework that conceptualizes safety and performance as emergent properties of interactions among people, tasks, technologies and tools, organizational conditions, the physical environment, and the external environment [33]. SEIPS 2.0 emphasizes person-centeredness and systems orientation, treating clinicians, patients, and caregivers as embedded components of work systems whose attributes interact with task demands and contextual constraints [35]. SEIPS 3.0 extends this foundation by reframing care as the patient journey distributed across encounters, settings, and organizations over time, highlighting transitions, interfaces, and adaptation as critical sources of both risk and resilience [36].

The Improving Diagnosis in Health Care report by the National Academies of Sciences, Engineering, and Medicine (NASEM) adopts and adapts SEIPS to focus specifically on diagnostic safety [9]. While maintaining SEIPS work system components, the NASEM framework explicitly shifts its focus toward diagnosis by framing diagnostic performance around accuracy, timeliness, and communication. It presents diagnosis as a collaborative and iterative process and highlights organizational learning and feedback as key mechanisms for improvement. In contrast to SEIPS’ design-oriented explanatory focus, the NASEM adaptation is more outcome-driven and normative, prioritizing where diagnostic processes fail and how systems should respond rather than systematically decomposing contributory mechanisms.

Building on both perspectives, our framework uses the SEIPS 3.0 structure to represent diagnosis as a distributed sociotechnical journey while explicitly aligning domains with diagnostic performance. Contributory factors are organized into six primary domains: Diagnostic Team Members, Tasks, Technologies and Tools, Organization, Physical Environment, and External Environment, with consideration for the dynamic interactions among these factors within a patient’s diagnostic journey, identifying obstacles to mitigate potential concerns and address in achieving accurate and timely diagnoses [36].

**Figure 1:**
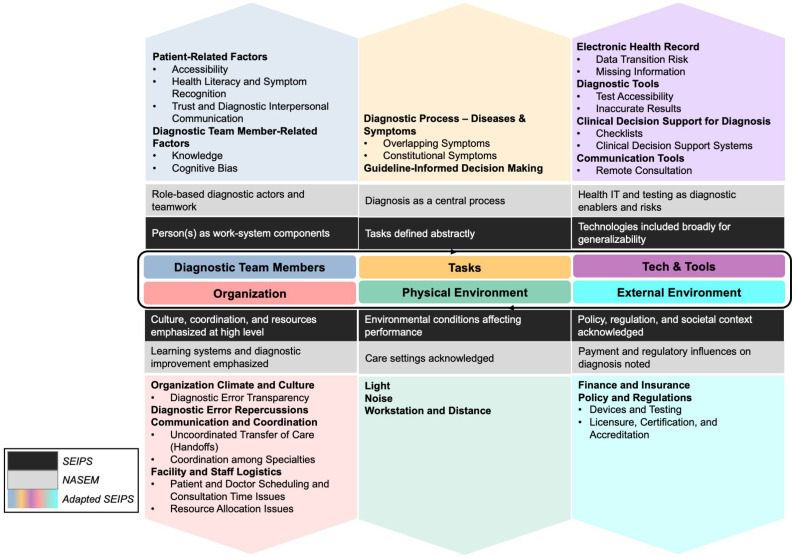
Diagnosis-Specific Literature Review Framework Adapted from SEIPS-NASEM models [9,36].

During literature synthesis, we identified diagnosis-relevant contributors that are conceptually acknowledged in SEIPS and the NASEM adaptation but are not explicitly delineated or consistently operationalized. SEIPS maintains high-level categories to preserve generalizability across healthcare work systems, while NASEM prioritizes diagnostic outcomes and improvement strategies over detailed mechanism specification. To address this gap, we operationalized each SEIPS domain at a lower level of abstraction by introducing diagnosis-specific subcategories that clarify how particular sociotechnical mechanisms influence diagnostic performance (Figure 1):

(1) Diagnostic Team Members: SEIPS conceptualizes “person(s)” broadly, and the NASEM framework emphasizes role-based diagnostic actors and teamwork. To add diagnostic specificity and analytic symmetry, we distinguished patient-related factors from diagnostic team member–related factors. Patient-related factors include Accessibility, Health Literacy and Symptom Recognition, and Trust and Diagnostic Interpersonal Communication, which directly shape the quality, completeness, and timing of diagnostic information entering the system. Diagnostic team member–related factors include Knowledge and Cognitive Bias that influence hypothesis formation, evidence interpretation, and follow-up decisions. By distinguishing patient-related and diagnostic team member–related contributors at this level, the framework extends SEIPS’ person-centered logic by adding the depth of diagnosis to the work system, explicitly modeling how information is generated, filtered, and acted upon by different actors within the diagnostic process.
(2) Tasks: SEIPS characterizes tasks using general attributes such as complexity and ambiguity, whereas NASEM treats diagnosis itself as the central process. To bridge this conceptual mismatch, we expanded the Tasks domain to include Diagnostic Process —Diseases & Symptoms, treating disease and symptom characteristics (e.g., Overlapping Symptoms and Constitutional Symptoms) as task inputs that systematically increase diagnostic uncertainty and cognitive workload. We also added Guideline-Informed Decision Making as a task-structuring element to capture how guideline definitions, exclusions, and constraints shape diagnostic decision making, particularly in the presence of comorbidities, atypical presentations, and unclear criteria for diagnostic accuracy or timeliness.
(3) Technologies and Tools: SEIPS addresses technologies broadly in terms of usability and fit, while NASEM emphasizes health information technology as both an enabler and a source of diagnostic risk. To reflect diagnosis-specific technological contributors while maintaining the SEIPS structure, we added Diagnostic Tests as a distinct subcategory to represent diagnostic tests and related technologies, including issues of Test Accessibility and Inaccurate Results. We also explicitly included Clinical Decision Support for Diagnosis, encompassing both Checklists and computerized Clinical Decision Support Systems (CDSS), to capture how decision aids can mitigate premature closure while also introducing risks related to automation bias, limited generalizability, and reduced transparency.
(4) Organization: SEIPS emphasizes culture, coordination, and resource constraints, and NASEM emphasizes learning systems and transparency. To connect these perspectives mechanistically, we added Diagnostic Error Repercussions to represent legal, reputational, and psychological consequences that influence clinician behavior, reporting, and defensive practices. We also explicitly delineated Organization Climate and Culture, including Diagnostic Error Transparency, hierarchical pressures, and peer norms that affect speaking up and feedback. In addition, we specified Communication and Coordination subcategories, including Uncoordinated Transfer of Care (handoffs) and Collaboration among Specialties, to capture how fragmented responsibility and information discontinuities contribute to diagnostic delay and error.
(5) Physical Environment: We retained SEIPS’ focus on environmental conditions (e.g., Lighting, Noise, Workstation Layout and Distance) but added diagnosis-relevant mechanisms that affect perception, attention, and examination quality.
(6) External Environment: Both SEIPS and NASEM acknowledge the influence of policy, regulation, and payment at a high level. We therefore added Policy and Regulations as an explicit subcategory to capture regulatory oversight, accreditation requirements, and standards shaping diagnostic tools, documentation, and permissible practice, alongside financial and insurance constraints affecting access, testing, and follow-up.

### 2.2. Review Approach and Justification

To effectively map diverse contributors within the SEIPS framework, a narrative review was selected to support the synthesis of system-level contributory factors to diagnostic errors. This approach enables critical interpretation and contextual integration rather than simple summarization [37,38], offering the flexibility to integrate a broad range of literature, including clinical, human-factors, organizational, and policy studies, which is critical for examining a complex, multidisciplinary topic [39].

### 2.3. Scope and Boundaries

To support the goal of identifying generalizable system contributors, this review was not restricted to a specific disease, specialty, or clinical setting. Instead, it encompassed factors influencing diagnostic accuracy, timeliness, and communication across inpatient and outpatient care, primary and specialty practice, and emergency and ambulatory environments.

Most included studies were published between 2000 and 2024, reflecting contemporary diagnostic workflows, digital infrastructure, and clinical decision-support environments. Earlier foundational work from 1956–1999 was incorporated selectively when it provided seminal theoretical grounding or clarified concepts not addressed in more recent literature. Only English-language publications were reviewed.

### 2.4. Literature Identification and Search Approach

Literature was identified using Google Scholar, with search guided by keyword combinations aligned with the SEIPS domains. Articles were eligible if they examined diagnostic error or contributory factors affecting diagnostic accuracy, timeliness, or communication. Eligible study types included observational research, qualitative studies, human factor analyses, conceptual papers, policy documents, and clinical guidelines. Search terms for each category are included in Table 1.

### 2.5. Contributory Factor Interactions

**Figure 2:**
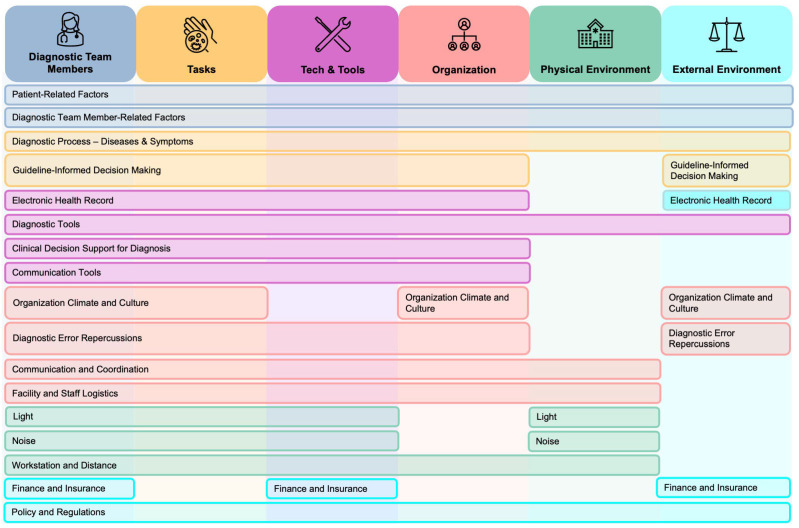
Interactions Between Key Contributory Factors across SEIPS Domains.

A key theme emerging from this review is that many contributory factors to diagnostic errors do not function within a single SEIPS domain but instead interact across domains and shape the diagnostic system as a whole (Figure 2). For example, limited patient health literacy influences not only communication with clinicians but also the accuracy of diagnostic tasks and the usability of technological tools. Similarly, communication quality extends across domains by affecting how clinicians gather information, build relationships with patients, and collaborate with team members within organizational workflows. Clinician cognitive biases intersect with organizational workflow pressures, such as short consultations and staffing constraints, as well as physical and environmental distractions, such as noise and layout. Likewise, workflow pressures further interact with task execution and clinician cognitive demand, reinforcing vulnerabilities across the system.

## 3. Diagnostic Team Members

### 3.1. Patient-Related Factors

Patient input is a valuable resource for diagnostic outcomes. Patient factors are multi-faceted and affect the quality and reliability of the input as well as the level of patient engagement. Logistical and healthcare resource accessibility affect the time and opportunity to get the first medical help. Health literacy determines a patient’s ability to recognize symptoms and understand the necessity to seek medical care. Furthermore, a patient’s trust and communication shape their relationship with clinicians, indicating their willingness to cooperate and share information.

#### 3.1.1. Accessibility

Logistical barriers are critical to achieving accessible care. Patients living in rural areas commonly have limited medical resources and staffing since advanced medical services and equipment are primarily found in urban areas or private facilities [40,41]. This makes access more difficult for those without reliable transportation or who face high travel costs [42,43]. This lack of nearby facilities can lead to longer delays compared with patients in urban areas, especially when facing rare diseases requiring specialized medical treatment and diagnosis [44]. Although telemedicine, including video calls and mobile consultations, offers an alternative for addressing the physician shortage in rural areas, the unstable and inadequate broadband infrastructure in these regions remains a significant obstacle to providing equitable care [45]. Patient relocation and mailing issues also hinder timely medical care delivery, as critical information such as regular or follow-up screening notifications may not be transmitted efficiently or accurately to the patients, increasing errors due to missing information [46]. For instance, during the COVID-19 pandemic, many patients moved without informing the clinical centers of their new contact details, making it difficult for the clinic to inform patients about abnormalities and schedule timely follow-ups [47]. Delays in referrals are also a common issue in the diagnostic process. Poor referral access can hinder patients’ opportunity for proper investigations, resulting in patients waiting longer or being sent to an unsuitable specialist [48,49]. For example, self-referred patients were found to experience 3.2 times more delay than those referred by their primary care providers (PCPs), as PCPs often play a crucial role in facilitating follow-up care and providing guidance and resources [50].

#### 3.1.2. Health Literacy & Symptom Recognition

Health literacy is defined as “the degree to which individuals can obtain, process, and understand basic health information and services needed to make appropriate health decisions,” [51]. Patient health literacy plays a crucial role in how accurately and quickly diagnoses are made. When individuals can comprehend health-related information, they are more likely to recognize early warning signs of disease. This understanding can help prevent diagnostic errors and receive timely and effective care [52,53]. For example, women with breast cancer who had self-examination knowledge were found to be more aware of any abnormalities and sought help from the physician earlier than those without that knowledge [25]. In addition to preventive screening and care-seeking behavior, health literacy impacts the management of diseases. A lack of knowledge about the disease can prevent patients from realizing the severity of their condition and seeking medical help until the symptoms worsen, especially for older adults. The mild symptoms in older adults often go untreated, as they often assume these are just a normal part of aging, thereby missing out on potential diagnoses [54].

Patients with low health literacy usually face obstacles in engaging with the healthcare system, including difficulties completing questionnaires [55], understanding clinicians’ professional terms and language [56,57], and knowing when to return for follow-up care [58,59]. Additionally, such patients are often more reluctant to seek medical care as they feel embarrassed by their inadequate health knowledge and avoid seeking assistance from others [60]. They might also pretend to understand the health-related document in front of their families to avoid shame, jeopardizing diagnosis accuracy and effectiveness of treatment outcomes [61].

#### 3.1.3. Trust and Diagnostic Interpersonal Communication

Patient-centered care is gaining more attention, shifting the patient-clinician relationship from focusing only on diagnosis to actively including patient opinions in discussions. This approach increases patient engagement and establishes a trusting relationship with providers, which enhances communication quality and encourages patient willingness to return for follow-ups [62]. However, some barriers, such as the patient’s previous experience, mental health, and cultural and social background, can hinder the development of trust and communication with clinicians during the diagnostic process [63]. Patients’ past negative experiences can hinder patient-provider communication and impair trust-building, making patients hesitate to engage in collaborative care [64,65]. For example, patients labeled as drug seekers by clinicians in previous visits may hesitate to return to the hospital due to the fear of being mistreated and misunderstood [66]. Additionally, some patients who perceived discrimination due to their race and gender reported feeling the service they received was rushed or "skipped over," complicating trust building and communication [67].

Patients with mental conditions face several barriers to communicating their symptoms. Affective and motivational problems can result in missed appointments [68]. Depression and psychotic conditions can cause poor concentration and disordered thinking, which can lead to inaccurate symptom histories. Anxiety and somatization disorders can also lead to exaggerated symptom histories inappropriately. In some cases, adherence to assessments that involve invasive examinations or procedures, such as blood draws and injections, or unfamiliar treatment settings, can be impaired by various mental conditions [69]. These obstacles complicate the diagnostic process, making it challenging for healthcare providers to acquire medical information from patients and take effective action [70].

Cultural and social disparities, including ethnic background and language barriers, can also influence how patients interact with providers [71,72]. For example, some patients demand specific testing methods or treatments due to their cultural considerations that are in conflict with current guidance from their healthcare providers [73]. Cultural communication styles may also differ, as shown in a study reporting that communication style differences between Turkish participants and their Dutch doctors often led to patient dissatisfaction. When doctors asked questions directly about their health problems, participants felt this approach was impersonal and careless, leading to their disengagement during the consultation [74].

Stigma often stems from value conflicts with societal norms, limited knowledge about diseases, and misconceptions about specific disease traits [75], which increases hesitation to seek medical help [76,77,78]. Patients often feel embarrassed or fearful about their diseases due to the stigma and worry about judgment from friends, family, or society [79]. For example, men are expected to display masculinity and independence in the social norm, which discourages them from pursuing mental health help [80]. In some religions, seeking sexual health care and having HIV/AIDS can be regarded as sinful and inappropriate, particularly for unmarried women [81]. Pressure from social norms and expectations results in the unwillingness to seek medical help and further deterioration of symptoms without proper and timely treatment.

### 3.2. Diagnostic Team Member-Related Factors

Clinician decision making plays a crucial role in determining how accurately and timely diagnoses are made. Clinicians depend on their expertise and experience as a foundation to analyze and understand key elements of patients’ conditions. If foundational knowledge or experience is insufficient, clinicians can potentially misinterpret or not recognize important diagnoses, which could lead to serious consequences. Additionally, bias can interfere with how clinicians evaluate and weigh risks and benefits, resulting in different tendencies for risk aversion or tolerance in their decision making. As a result, this may cause diagnostic errors due to over- and under-testing, or inappropriate decisions regarding the timeliness and intensity of monitoring and care, such as whether patients require inpatient or outpatient management, or the frequency of follow-up schedules.

#### 3.2.1. Knowledge

A clinician’s professional knowledge and previous experience can significantly influence symptom interpretation and diagnosis. Greater diagnostic knowledge, including the ability to analyze clinical data, make accurate differential diagnoses, and use diagnostic tests effectively, is associated with fewer adverse outcomes among high-risk diagnostic error situations [82]. In contrast, clinicians with outdated or inadequate knowledge often fail to identify diseases and symptoms in their early stages and may misinterpret clinical guidelines [83]. Clinicians may choose to excessively or inappropriately rely on their instinctual judgment instead of established diagnostic criteria, which can result in inconsistent care [84] and increase morbidity and mortality [85].

Expertise varies across medical specialties, with primary care physicians (PCPs) having a broad expertise to provide comprehensive healthcare services, often serving as the first point of contact for a patient with a complex medical condition [86]. In contrast, specialists have expertise that focuses on a single, narrowly defined discipline, commonly receiving referrals from PCPs for patients who require more specialized care. Diagnostic errors can manifest in different ways among clinicians, depending on their specialty. For example, specialists who are more familiar with disease-specific diagnostic guidelines may conduct more comprehensive examinations and may detect disease at an earlier stage [87]. However, over-specialized knowledge is sometimes a double-edged sword during diagnosis, limiting diagnostic possibilities and leading to neglect of factors outside of the specialty, especially in complex symptom presentations [88].

#### 3.2.2. Cognitive Bias

Systematic errors are consistent or predictable inaccuracies in measurement, often arising from theoretical and assumption errors, observer bias, and calibration issues [89]. Cognitive bias is a common systematic error that influences the human decision-making process by affecting how information is interpreted or tasks are prioritized. Kahneman (2011) introduced the concept of two systems of thinking in “Thinking, Fast and Slow”: System 1, which is fast, intuitive, and emotional, and System 2, which is slow, conscious, and analytical [90]. In clinical settings, clinicians often tackle tasks using System 1 thinking, particularly in time-sensitive or high-pressure situations [91]. Though this helps facilitate rapid decision making by using heuristics or mental shortcuts to reduce cognitive workload, it can also lead to judgment errors and cognitive biases [92,93], such as anchoring, confirmation, attribution, availability, confidence, and delay discounting. Such biases prevent clinicians from exploring more information and considering alternative diagnoses, leading to incomplete or insufficient assessments [94]. However, not all heuristics result in poor decisions. Gigerenzer and Brighton (2012) suggest that simple heuristics enable individuals to make accurate and efficient choices with limited yet relevant information, proving effective and reducing errors in real-world, uncertain environments like healthcare [95].

Anchoring is defined as the process in which individuals rely heavily on an initial value or information (the “anchor”) when making decisions or estimates. The adjustments after the initial value, or the starting point, are usually insufficient, yielding biased judgments [96]. Clinicians exihit this bias by placing excessive weight on the initial information they receive or salient symptom features a patient presents, focusing on confirming their initial assumption during the diagnostic process and concluding the diagnosis without thoroughly investigating other alternative possibilities [97,98]. Information such as pre-existing notes from prior consultants, normal imaging results, and the presence of yellow flags directs clinicians to focus on their original diagnostic hypothesis without further questioning secondary or alternative possibilities [99].

Attribution, as explained in Fritz Heider’s Attribution Theory, refers to how individuals perceive and interpret the causes of behaviors and events, attributing them either to internal (dispositional) factors, such as emotions, talents, and personal characteristics, or to external (situational) factors, such as environmental influences [100]. Attribution bias in clinicians occurs when their decision making and judgment are influenced by patients’ traits or their stereotypes toward patients, which mislead the diagnostic process and result in errors [97]. Patient information such as age, name, sex, and ethnicity can distract clinicians by leading them to focus on these characteristics rather than objectively evaluating the patient’s symptoms and physical conditions. For example, when diagnosing older patients, symptoms such as heart failure may only be attributed to being old rather than other possible reasons [54].

Availability bias occurs when individuals assess the probability of an event based on how easily examples come to mind, which may not accurately reflect actual frequency or risk [101]. Consequently, clinicians may overestimate the frequency of a diagnosis if a case or condition comes to mind quickly and easily, even if it is less common. For example, when there are recent or notable cases, clinicians tend to make a diagnosis based on these events, often overlooking other factors that may be more relevant or more closely aligned with the patient’s symptoms [102].

Confidence bias, including overconfidence or underconfidence, also plays a significant role in shaping clinical decision making and can lead to critical errors. Overconfidence is one of the most common biases in the clinical setting [103], as clinicians sometimes trust their own judgment more than engaging in thorough investigations. For example, a study demonstrated that participants in the high-confidence group tended to overestimate their diagnostic accuracy, even though their actual performance was similar to that of the low-confidence group [104]. Overconfidence leads to quicker, less thorough diagnostic evaluations, increasing the risk of errors by prematurely closing the diagnostic process and failing to consider alternative possibilities [105]. On the other hand, underconfident clinicians are likely to seek external information to validate and ensure their diagnostic accuracy [106,107], resulting in over testing and delays in decision making [108].

Delay discounting refers to the decision making process in which an individual prefers between selecting an immediate reward with less value and waiting longer for a more significant value [109]. For example, an experiment in Yeh et al.’s study (2021) asked participants to choose between an immediate reward of $100 today or $200 in 1 month [110]. Clinicians’ decision making in complex cases mirrors delay discounting, as they balance their perceived best benefits with the relative utility of possible health outcomes to effectively weigh risks and benefits [111]. This bias is often exacerbated by “sludge” or the frictions or administrative burdens that make it difficult for people to achieve what they want or need [28,112]. The scarcity of clinicians’ time creates cognitive pressure, which leads to decisions favoring immediate efficiency rather than focusing on long-term clinical value and outcomes. As a result, clinicians often sacrifice the time spent with patients to commit to the required administrative duties [29,113,114]. The excessive clerical tasks not only interrupt clinicians’ critical thinking about diagnostic evaluation [115] but also compress patient interaction. Limited interaction impedes comprehensive information collection and patient observation, potentially increasing miscommunication [116]. Insufficient communication between patients and clinicians causes clinicians to make diagnoses without further evaluation and leads to diagnostic errors and low clinical quality [117]. The excessive workload also contributes to clinicians’ burnout [29,30], fatigue [118,119], and emotional exhaustion [120,121].

## 4. Tasks

The characteristics of the disease, including the patient’s symptoms, often add to the challenges of achieving accurate and timely diagnoses. Overlapping and constitutional symptoms are likely to mislead clinicians, increasing the risk of misinterpretation or failure to identify the underlying diseases. Diagnostic guidelines offer frameworks and criteria for clinicians to follow in a more structured way. However, some gaps in the guidelines allow for open interpretation. The inconsistency among different subjective judgments makes both clinicians and patients vulnerable to diagnostic errors.

### 4.1. Diagnostic Process—Diseases & Symptoms

#### 4.1.1. Overlapping Symptoms

Overlapping symptoms associated with various diseases are a major contributor to diagnostic errors [122,123]. Clinicians face considerable difficulties identifying and differentiating between similar conditions, especially when symptoms frequently appear across many common diseases [124,125]. Symptoms such as dyspnea and wheezing, which are commonly linked to chronic obstructive pulmonary disease (COPD), can also occur in other conditions like congestive heart failure (CHF), asthma, lung cancer, pneumonia, and interstitial lung diseases [126,127]. Sometimes, overlapping symptoms can be hard to differentiate, particularly during advanced diagnostic assessments [128]. For example, research shows that overlapping findings in amyotrophic lateral sclerosis (ALS) and other neuromuscular disorders assessed through electromyography (EMG) still need further testing and the engagement of a multidisciplinary team for interpretation to ensure an accurate diagnosis [52]. Furthermore, the variety of characteristics seen in different diseases adds a layer of complexity to the diagnostic process [129,130]. The wide range of symptoms and behaviors, accompanied by different severity and combination levels, complicates diagnosis [131,132]. For example, this variability is especially common in autism spectrum disorder (ASD), which frequently exhibits symptoms that overlap with other psychiatric conditions. The common characteristics of ASD, like social isolation, diminished social insight, and repetitive behaviors, can also be found in other psychiatric disorders, such as schizotypal personality disorder and psychotic disorders, leading clinicians to easily mistake the signs for other disorders [20].

#### 4.1.2. Constitutional Symptoms

Constitutional or non-specific symptoms are general symptoms that offer little diagnostic value and are not exclusive to any specific disease or condition, as they can arise from multiple causes, leading to issues in differentiating possible diagnoses [133]. When patients arrive at the clinic, they are evaluated and triaged by their symptoms [134]. Patients with site-specific symptoms, located in a particular area of the body, are more likely to raise the “alarm” for clinicians regarding urgent referrals [135]. In contrast, patients presenting non-specific symptoms, such as weight loss or fatigue, are typically regarded as having non-serious conditions and are more likely to experience diagnostic delays than site-specific patients [136]. For example, children with bone tumors often present non-specific symptoms, such as pain or fatigue, which can be easily labeled as minor or common concerns. On the other hand, noticeable symptoms, like a visible mass, generally result in more prompt referrals and quicker diagnoses [137].

Furthermore, applying specific diagnostic tests to the general population with constitutional symptoms can result in low-value outcomes. For example, vitamin D screening is intended to identify vitamin D deficiency in at-risk individuals. However, it is often ordered for patients with general symptoms like fatigue or vague pain, rather than those who are actually at risk. Research shows that detecting and treating mild deficiencies has a minimal impact on symptoms or overall health outcomes but can lead to unnecessary testing and increased costs [138].

### 4.2. Guideline-Informed Decision Making

Diagnostic guidelines provide a standardized and evidence-based framework that enhances accuracy, consistency, and quality in diagnosis, ultimately supporting more efficient and reliable care and improved patient outcomes [139,140]. However, many diagnostic guidelines are incomplete and have several limitations, including a lack of clear definitions [141,142], inadequate consideration for comorbidities [143,144], and lacking standardized testing methods [145,146].

The report, Improving Diagnosis in Health Care by NASEM, defined a diagnostic error as the failure to establish an accurate and timely explanation of the patient’s health problem(s) or communicate that explanation to the patient [9]. However, the report did not clearly specify the criteria for what constitutes an accurate, timely, and well-communicated diagnosis. In the years after its release, while there has been a marked increase in scholarly discussions regarding diagnostic errors, most of these discussion lack a clear definition of diagnostic error. For example, the College of American Pathologists (CAP) guidelines recommend reviewing patient cases to reduce interpretive diagnostic errors in surgical pathology and cytology [147]. However, clear benchmarks for timeliness were not provided. The guidelines stated that case reviews should be performed in a timely manner but didn’t specify how quickly they should be conducted to align with clinical workflow. Furthermore, the CAP guidelines prioritize error detection through secondary reviews without providing quantifiable metrics for measuring diagnostic accuracy. This highlights the difficulty in creating universal standards for diagnostic quality.

In addition to the aforementioned issues with diagnostic error definitions, most clinical practice guidelines target specific diseases, ignoring their relevance for patients with various comorbidities [148,149,150]. More than a quarter (27.2%) of adults in the US have multiple chronic conditions [151]. Yet, many guidelines fail to address the practical implementation for clinicians and the needs of patients with multiple chronic conditions [152]. When multiple guidelines are used simultaneously, patients will experience cumulative treatment burdens. For instance, the National Institute for Health and Clinical Excellence (NICE) guidelines offer recommendations on diagnosis, treatment, prevention, and management of various medical conditions for doctors in the United Kingdom [153]. However, guidelines for individual diseases often do not account for multimorbidity, leading to duplicate testing and a higher treatment burden [154]. If clinicians were to adhere strictly to every NICE guideline for specific diseases, patients would face numerous appointments and be prescribed excessive medication [155]. The absence of guidance on prioritizing treatment plans for patients with multiple conditions makes the care process overwhelming and difficult to manage.

The lack of standardized procedures in guidelines also complicates diagnostic testing and increases the risk of delays. This is particularly true for emerging diseases, where limited data and evolving traits make consistency difficult [156]. The inconsistent recommendations primarily originate from connections with unclear underlying evidence [146,157]. For instance, at the beginning of COVID-19, diagnostic methods for respiratory assessments differed significantly among nations and lacked uniform implementation. Furthermore, the disease’s tendency for false negatives often requires retesting, but the lack of recommended time intervals between tests in the guidelines leads to potential diagnostic delays [158]. This issue is also present in cancer diagnosis, such as with well-differentiated liposarcoma. The inconsistent application of diagnostic criteria across institutions and among pathologists results in significant variability in interpretation and diagnosis, and these issues often remain unnoticed without secondary reviews [159].

To address these challenges, standardized systems and procedures have been developed to overcome and reduce the variation in diagnosis. For example, the Bethesda System (TBS) for Reporting Cervical Cytology was created to reduce variation in Pap test interpretation by standardizing terminology, ensuring consistent test results across labs, and recommending appropriate next steps in care [160]. Similarly, the Reporting and Data Systems (RADS) were developed to standardize imaging reports for various organs, especially focusing on cancer detection, providing structured reporting formats and risk stratification to improve communication between radiologists and referring clinicians [161,162].

## 5. Technologies and Tools

### 5.1. Electronic Health Record (EHR)

EHRs are important clinical data repositories for patient medical history, family history, and lab results, enabling clinicians to conduct precise and well-informed diagnoses [163]. They provide clinicians with timely access to critical clinical data structured in ways that enhance diagnostic workflows and support accurate decision making [164]. However, EHRs potentially contribute to over 60% of diagnostic errors in ambulatory settings [165]. Significant issues such as incomplete data during data migration, missing information in EHRs, and insufficient functionality lead to diagnostic errors.

#### 5.1.1. Data Transition Risk

There is an increasing amount of hospitals switching their EHR systems to other vendors to improve workflow efficiency, regulatory compliance, and data sharing to achieve cost-effectiveness [166]. However, challenges, such as system construction variability, missing data, and dependence on original systems, lead to conversion errors such as corrupted data and mismatched patient records [167], disrupting clinical workflows and compromising patient safety [168]. A key challenge in EHR systems is their contextual variability, with significant differences in documentation styles and data structures across institutions that impact the types of information recorded [169]. For example, anemia is named differently across systems; one might call it “post-hemorrhagic anemia”, while another refers to it as “blood loss anemia”. Such inconsistencies result in patient record discrepancies and complicate clinician interpretations [170]. Another issue in migrating EHR data is the free text input. While clinicians frequently add critical clinical information in this section, the unorganized formats and the presence of irrelevant or unclear details complicate data transfer due to inconsistencies and variability [171]. For example, free-text allergy information in EHRs, such as “Doesn’t remember the drug,” “Begins with a C,” or “Almost all antibiotics,” offers limited insight into potential allergens, hindering clinicians’ access to precise and comprehensive diagnostic information and potentially obstructing accurate diagnoses [172]. This challenge is especially common since many systems only use proprietary vocabularies that lack compatibility [173].

#### 5.1.2. Missing Information

Documentation errors, especially data omissions, can influence diagnostic decisions and lead to inappropriate treatments [174,175]. Earlier diagnosis and fewer redundant investigations can be achieved using comprehensive and precise patient information [176]. However, 71.5% of medical errors arise from missing information about patient symptoms or medical history details [177]. For example, studies on cancer registries reveal frequent missing data due to inadequate documentation, such as absent cancer stage or test results, which affects the diagnosis and evaluation process and lowers survival rates [178]. Additionally, interoperability problems when transferring data between two different medical record systems can result in absent final diagnoses or national diagnostic codes, compromising the quality of the information and leading to diagnostic errors [179]. Another contributing factor to missing data is the "copy-paste" function in EHRs, which allows clinicians to reuse existing medical notes without carefully reviewing them again [180,181]. This practice is commonly seen in busy clinical settings where time pressure is imposed on clinicians to complete documentation quickly [182]. Unfortunately, this shortcut results in inaccuracies, outdated information, and sometimes misleading patient records [183]. These problems result in "note bloat," which buries key diagnostic information under unnecessary documentation and limiting clinicians’ capacity to reevaluate and adjust diagnoses as conditions change [183]. Patients’ conditions may be updated inaccurately if clinicians heavily rely on the copy-paste function, raising the risk of errors in the following patient care [184].

### 5.2. Diagnostic Tests

Laboratory tests provide analytical results that support diagnostic processes and disease management. Clinicians can achieve timely and accurate diagnoses using appropriate tests and resources. However, when the critical tests are unavailable, clinicians will have to seek alternative options or make diagnoses based on their judgment instead. Logistical constraints and resource shortage issues prolong the diagnosis time. Inaccuracy in test results, such as false positives and negatives, also misleads clinicians with seemingly plausible outcomes.

#### 5.2.1. Test Accessibility

There are two primary accessibility issues in diagnostic testing that contribute to errors in diagnosis. First, there is a lack of appropriate or specific diagnostic tests for certain conditions or diseases, especially in their early stages [185,186,187]. Without definitive tests, healthcare providers often depend on clinical judgment and broader testing approaches, sometimes resulting in diagnostic errors. For instance, there is no definitive diagnostic test for irritable bowel syndrome (IBS). The condition is primarily diagnosed based on symptom criteria and the exclusion of other gastrointestinal disorders. The uncertainty caused by overlapping symptoms with other conditions and the lack of an objective biomarker can lead to potential diagnostic challenges [188].

Second, availability issues, such as geographical constraints, resource shortages, or long waiting times, can cause significant diagnostic errors even when specific tests exist [189,190]. More advanced diagnostic equipment is typically found in higher-tier facilities, and the referral process prolongs the time before patients receive diagnosis and care [191]. For example, in heart failure, tests like B-type natriuretic peptide (BNP) and echocardiograms are less accessible in rural or underserved healthcare settings, leading to delayed diagnoses and treatments [54].

#### 5.2.2. Inaccurate Results

Inaccurate diagnostic test results primarily manifest as two error types: false positives and false negatives [192]. False positives can lead to unnecessary examinations and treatments, while false negatives may result in missed diagnoses and intervention delays. Both types of errors undermine the reliability of diagnostic tests for patients and clinicians, reducing overall trust in the diagnostic process [193].

A false positive occurs when a test mistakenly shows that a condition or disease exists when it actually does not [194]. In some cases, false positives can lead to unnecessary treatments, psychological distress, additional testing, and occasionally unwarranted invasive procedures [192]. For example, false positives in breast cancer diagnoses often arise from misclassifying benign lesions like atypical ductal hyperplasia as invasive cancer, leading to unnecessary and costly treatments such as surgery, radiation, or chemotherapy [195].

On the other hand, a false-negative test result occurs when a diagnostic test does not detect a present condition [194]. This oversight harms patient outcomes and weakens trust in diagnostic accuracy, fostering a false sense of security and postponing essential interventions [192]. For example, computed tomography (CT) urography, often used for initial bladder cancer screening, has a high false negative rate due to technical limitations in detecting small or flat lesions and obstructions from residual urine or surgical artifacts. Therefore, cystoscopy is automatically included in the diagnosis process to ensure accurate diagnosis and appropriate follow-up. Similarly, in malaria-endemic regions, rapid diagnostic tests (RDTs) can yield false negatives for reasons such as low parasite density and improper storage of RDTs, resulting in approximately 20% of microscopy-confirmed malaria cases being missed [196].

When a single positive or negative threshold for a test is unclear, the receiver operating characteristic (ROC) curve, a plot of sensitivity against specificity across all possible cutpoints, is particularly useful for continuous or ordinal results [197]. However, since ROC curves use the average performance across all thresholds, including those not used in clinical practice, this can limit their clinical relevance. Additionally, because ROC analysis doesn’t consider disease prevalence, it can lead to interpretation bias and may not accurately reflect real-world settings [198,199].

### 5.3. Clinical Decision Support for Diagnosis

Clinical decision support encompasses a range of tools used to enhance diagnostic safety in clinical settings. These include checklists, which may be implemented in paper or digital forms and software-based clinical decision support systems (CDSSs).

#### 5.3.1. Checklists

Clinical checklists turn evidence-based guidelines into clear, practical steps that reduce confusion and help ensure that every patient receives safe, consistent, and high-quality care [200,201]. Checklists are designed to guide diagnostic reasoning and act as cognitive aids that support clinicians. They encourage analytic thinking instead of relying solely on intuition and memory, prompting them to consciously examine their diagnostic approach [202,203]. They also assist clinicians in considering a broader range of potential factors and encourage a more comprehensive review of important items and variables [204,205]. The structured approach helps identify errors, correct them, and prevent premature closure [206]. For example, the TWED (Threat, What else, Evidence, Dispositional factors) checklist prompts doctors to question their initial diagnosis and consider possible influencing factors, ensure there is enough supporting evidence, and explore alternative possibilities instead of settling on the obvious initial diagnosis [207]. However, while checklists can reduce diagnostic errors, the drawback of checklists has also been found to interrupt expert-level reasoning strategies in the early stage of the diagnosis process and lead to reduced performance [208]. Moreover, if poorly designed or not implemented rigorously, checklists may limit clinical flexibility and fail to accommodate the complexity of real-world diagnostic reasoning [209]. Each medical environment has distinct clinical requirements and needs a customized checklist that fits the team’s workflow and structure [210].

#### 5.3.2. Clinical Decision Support Systems (CDSS)

CDSS are software tools designed to help healthcare professionals make clinical decisions by integrating patient-specific information with a knowledge database using advanced algorithms to deliver customized recommendations or assessments in real time [211]. The goal of the system is not to replace clinicians but to aid them in complex data and scenarios by combining clinical knowledge and technologies, thereby securing diagnostic performance and delivering optimized care [212,213].

By integrating and analyzing patient symptoms, medical history, test results, and other risk factors, CDSS have the capability to support disease detection and identification [214,215], such as the subtle or ambiguous patterns that clinicians often overlook [216,217]. For example, a study developed a CDSS for cardiovascular diseases by integrating multimodal data from hospital records, tests, and electrocardiograms (ECGs) with clinical guidelines and risk models. When clinicians entered orders or recorded patient information, the system automatically recommended diagnostic or therapeutic actions [218]. Some CDSS can further generate ranked lists of potential diagnoses for disease differentiation [219,220,221], including disease subtype classification or stages, to guide subsequent treatment planning [222]. Artificial intelligence (AI) can further expand CDSS capabilities beyond simply disease differentiation and be used to predict treatment response [223]. For example, a study created a CDSS for gastric cancer that used deep learning trained on an endoscopic image dataset. When clinicians performed procedures, the system supported real-time detection by highlighting suspicious areas and classifying disease stages to inform diagnosis and treatment decisions [222]. Additionally, some CDSS can track disease progression, predict outcomes, and support follow-up planning [224,225]. For example, CDSS applied in chronic kidney disease (CKD) can simulate disease progression and predict adverse events when medication or lifestyle changes are made, guiding long-term patient management [225].

Although specialized and domain-specific CDSS have been successfully implemented in several fields, the overall effectiveness of CDSS still needs further validation and improvement [164]. In many cases, the accuracy of CDSS is often questioned due to methodological limitations, unrepresentative patient samples, and insufficient external validation, all of which undermine reliability and generalizability [226]. For example, in the diagnosis of leukemia, a CDSS must incorporate the combined verification of both blood cell counts and bone marrow test results. However, a poorly designed system that relies on only one input and fails to account for this joint necessity risks producing inaccurate predictions and unsafe recommendations [227]. Furthermore, AI-driven CDSS introduce challenges. If only certain populations are included in the dataset, some systems may be trained with biased data and generate unrepresentative outcomes that can further exacerbate health inequities [228,229]. Clinicians may also become over-reliant on automation, placing excessive trust in system recommendations and neglecting their own clinical judgment, which increases the risk of errors when the system’s recommendations are incorrect [230,231]. Other observed challenges in various CDSS include static and non-updatable knowledge bases, which restrict the systems’ ability to provide the most current and relevant care [232]. Additionally, the lack of transparency in AI-based and complex CDSS compared to rule-based systems often makes their errors harder for clinicians to predict and understand. This can impact trust between clinicians and the system and may even lead to doubting the system’s output [233].

### 5.4. Communication Tools

#### Remote Consultation

Remote consultation in telemedicine allows both patients and providers to access care and exchange medical information via phone or video, regardless of their distance [234,235]. In recent years, remote consultations have been utilized to provide a safer alternative option for patients and clinicians while also preventing the spread of infections in the clinical space [236]. Vulnerable populations, including racial and ethnic minorities and older adults, can benefit from virtual visits that enable more equitable access to healthcare services [237]. However, digital limitations can constrain clinical information gathering, affecting the quality of care and the accuracy of diagnosis.

Physical examinations are the major constraint in remote consultation. The inability to palpate or acquire certain physical signs without advanced equipment can impede diagnostic judgment due to missed details [238,239]. The physical examination is critical in supporting diagnoses and is required in nearly 80% of in-person general practitioner (GP) consultations [240]. The lack of remote physical exams, like abdominal assessments and vital checks, can create significant challenges in telemedicine, as essential clinical signals can be missed in video or phone consultations [241]. Moreover, non-verbal cues, such as facial expressions, body language, and subtle symptom presentation, can be ignored or unobserved during remote consultation. The missing detailed observations may be crucial for diagnoses [242].

Another challenge of remote consultations stems from infrastructure and device technical issues [234,243]. The different quality and functionality of patients’ devices, such as camera resolutions, can impact the information quality gathered in remote consultation [244]. Device limitations can challenge the accuracy and effectiveness of a self-examination [245]. For example, patients might struggle to show the clinician the required viewing angle while following the instructions through video calls or experience delayed responses. As a result, the accuracy and quality of the diagnostic examination can be inconsistent [246].

## 6. Organization

### 6.1. Organization Climate and Culture

#### Diagnostic Error Transparency

An ideal organizational culture is a supportive environment where clinicians are encouraged to share, discuss, and learn from mistakes without any concerns, which is essential for handling diagnostic errors stemming from cognitive and systemic inefficiencies [247]. However, an organizational culture can be influenced by the fear of internal hierarchies, peer pressure, and loss of professional reputation that can inhibit clinicians from openly discussing mistakes, hindering communication and opportunities to improve performance.

A fear of hierarchies can stem from power imbalances [248,249] and cultural taboos [250] between senior and junior staff. Junior clinicians may hesitate to report patient safety issues to avoid conflict with their senior leaders, fearing this can jeopardize their future careers. This reluctance creates communication gaps, resulting in missed chances for valuable feedback and timely interventions [31]. Despite the heavy reliance on teamwork in healthcare, ambiguous roles and significant power imbalances among team members can lead to differing levels of involvement and create feelings of exclusion, especially for those in subordinate positions [251]. In inpatient multidisciplinary healthcare teams, excluding direct care nurses and assistants from the core decision making team can lead to alienation. Nurses and assistants may feel that their opinions are not acknowledged, leading to their unwillingness to follow and engage with directives [252]. Pressure from social norms and shaming can inhibit clinicians from reporting or discussing clinical errors, as it may result in judgments about their competency and damage their reputation [32,253]. Moreover, due to the fear of undermining professional relationships, specialists frequently do not report diagnostic errors. For example, when a specialist discovers an error, they often avoid reporting it to the original specialist or making referrals about it [254,255]. This can result in challenges when incorporating diverse opinions among a healthcare team, which are necessary to create a thorough diagnosis [25], and hinder the opportunity to learn from feedback that enhances mistake prevention and patient care [256].

### 6.2. Diagnostic Error Repercussions

The medical malpractice system and state licensing boards exist to reinforce the standards and code of conduct for medical professionals in order to protect public health. However, they can also cause healthcare staff to be less willing to report medical errors due to the fear of adverse administrative consequences, suggesting the importance of creating a tolerant and safe working culture that emphasizes learning [257,258]. Common consequences for diagnostic errors are error and warning records in the clinicians’ personnel files [141], revocation or restriction of license [259], probation, and suspension [260].

Clinicians also face malpractice claims for adverse events. Payments for diagnostic errors attributed to malpractice are reported as one of the most common categories of claim, accounting for 21% of the payments. This is primarily due to the difficulties in recognizing subtle symptoms during a single outpatient visit, making follow-up and accurate diagnosis more difficult [261]. Clinicians’ decision making can be greatly impacted by the potential medical liability risk, which often leads to selecting defensive practices, such as ordering unnecessary tests and referrals, that are perceived as mitigating malpractice claim risk instead of clinically necessary actions to avoid legal issues [262,263,264]. For example, due to the fear of medical liability, some radiologists may select defensive behaviors that conclude the ambiguous image finding is abnormal [265]. Although these practices are often perceived as legally protective, they can delay diagnoses, increase errors, raise healthcare expenses, and undermine patient outcomes by diverting attention from timely clinical decision making [266].

### 6.3. Communication and Coordination

#### 6.3.1. Uncoordinated Transfer of Care (Handoffs)

Handoffs involve transferring responsibility, information, or tasks between individuals, teams, or departments. Factors such as time constraints, interruptions, unclear terminology, language barriers, cultural aspects, and poor documentation lead to overlooked information and improper transfers [267,268]. Poor handoff can cause several risks, including loss of crucial information that may result in diagnostic errors, and delays in pending tests and results [269]. Communication problems during handoffs occur in various environments, particularly frequently during patient transfers from external facilities, where incomplete information is commonly received [25].

The quality of information may significantly decline or be omitted during the numerous handoff communications. More than 87% of ICU doctors indicated that they faced adverse events related to communication issues during handoffs, such as omissions or misunderstandings of crucial diagnostic details, including pending results and required follow-up care [270]. The increasing complexity of transitions in healthcare can easily result in poor handover, which undermines care continuity and diagnostic procedures [269]. Moreover, this problem can go beyond personal communication; inconsistencies arise between verbal handoffs and EHRs. Errors and gaps may happen when clinicians verbally report a test result differently from what is recorded in the medical files, as well as when critical information from lab results is left out during handoff discussions [271]. These issues contribute to a knowledge gap, resulting in misunderstandings about patient conditions, unnecessary tests, and delays in treatment.

#### 6.3.2. Collaboration Among Specialties

Diagnostic errors often occur when multiple specialists are involved in the diagnostic process. When a patient’s condition presents with ambiguous or overlapping symptoms, which can be difficult to distinguish from one another, this may lead to consultations with different specialists and referrals to arrive at a final diagnosis [272,273]. This can result in unnecessary tests, delays, and misdiagnoses as patients are referred between specialists without a unified approach to diagnosis [88,274]. For example, patients with IgG4-related disease (IgG4-RD), a rare inflammatory condition with symptoms associated with cancers or autoimmune disorders, ultimately often consult multiple specialists. Before an accurate diagnosis is made, unnecessary surgeries or treatments are usually conducted due to multiple referrals for misdiagnosed conditions [274]. Patients with head-and-neck cancer may also need to consult with various specialists due to the symptoms resembling benign conditions. This fragmented care pathway frequently results in prolonged diagnostic delays, mismanagement, and extended waits for essential tests before reaching an accurate diagnosis [275].

### 6.4. Facility and Staff Logistics

#### 6.4.1. Patient & Doctor Scheduling and Consultation Time Issues

Inefficiencies in appointment scheduling and short consultation times pose a significant obstacle to prompt diagnosis and frequently result in considerable delays in patient care. Patients face a number of difficulties when booking appointments in healthcare settings due to the inconsistent nature of service times, common no-shows, and an imbalance between supply and demand, leading to extended wait times and wasted resources.

Scheduling appointments poses significant challenges in primary care and postpones critical diagnoses. Given the mismatch between available time slots, clinician schedules, and patient needs, clinics often find it challenging to meet patients’ expectations, especially during busy periods [20,276,277]. To address the issues resulting from no-shows and last-minute cancelations, using overbooking as a solution often causes an increase in patients’ wait time and clinicians’ overtime [278]. Furthermore, the scheduling systems were initially aimed to minimize idle time but failed to adapt to the unpredictable nature of the healthcare system [277]. The scarcity of availability and long wait times hinder patients from accessing timely evaluations, and their conditions may deteriorate before being identified and treated [279,280]. Additionally, clinicians may have difficulty staying vigilant under the heavy workload [25,281]. In order to accommodate high patient demand, clinicians will have to shorten consultation times [276]. Limited consultation times restrict clinicians from conducting thorough evaluations, such as more advanced assessments and interactions with patients, and often lead to poor decision making [282]. Therefore, clinicians often focus more on common or easily treatable conditions, potentially overlooking subtle diagnostic signs or symptoms due to time pressure. This results in delays in addressing critical underlying issues and diminishes the quality of care [54]. Longer consultations allow for a more thorough assessment, which helps improve diagnoses and reduces the chance of missing important details [283,284,285].

Clinician diagnostic performance is likely to vary depending on the time of day. A study on skin biopsies found that dermatologists’ accuracy tends to decrease toward the end of the day, especially on Fridays. Fatigue from repetitive procedures or decision-making can impair diagnostic accuracy, making clinicians more prone to identify diseases that are not actually present [286].

#### 6.4.2. Resource Allocation Issues

Most healthcare service delays are defined as the time between patients’ first arrival at the facility and their completion of evaluations and receipt of diagnostic results [287,288]. Laboratory testing is critical for determining diagnoses, treatments, and discharge decisions. However, late morning to early afternoon tends to be the busiest time, and the congestion often leads to longer turnaround times, especially when insufficient staffing exists [289]. The allocation of diagnostic tools such as CT and MRI frequently faces obstacles due to inflexible scheduling slots. The capacity is underutilized during low demand, while in busy periods, non-emergency patients often experience delays [290]. Poor equipment and facility allocation can also lead to a bottleneck, hindering patient flow and decreasing clinician utilization. For example, inefficient exam room allocation makes clinicians spend time waiting for availability, which reduces the number of patients they can see [291]. Bed allocation also presents a similar problem. Even though hospitals often aim to maintain 85% bed occupancy, they still face congestion as patient needs change unexpectedly or patients are not properly distributed across the departments [292]. During periods of low demand, capacity may be underused, while in busy times, non-urgent patients experience delays. This imbalance can disrupt hospital workflows and contribute to diagnostic errors.

## 7. Physical Environment

The physical environment, including lighting, noise, and spatial arrangement, is directly related to clinicians’ performance by reducing cognitive load, limiting distractions, and enhancing comfort—each essential for maintaining accuracy and efficiency in high-pressure healthcare environments [293].

### 7.1. Light

Poor lighting conditions and quality cause discomfort and eye strain, making it difficult to concentrate on visually demanding tasks difficult and leading to misunderstandings and misinterpretations of critical diagnostic information [293]. Lighting conditions like flickering, glare, and distracting shadows can induce visual discomfort. Constant eye strain and posture adjustments to see details more clearly will complicate and prolong the diagnostic process [294]. Moreover, inadequate lighting in critical areas is associated with reduced observation accuracy, which may contribute to diagnostic errors. For example, radiologists rely on proper ambient lighting to balance image luminance and minimize glare. Constant eye adjustments between a bright screen and a dark environment can cause eye fatigue and potentially affect information interpretation [295]. The importance of lighting quality can also be seen in dermatology, as it affects the accurate display of skin tones essential for diagnosing conditions like anemia or jaundice. Poor lighting limits the clinician’s capacity to conduct subtle skin assessments, frustrating dermatologists and compromising diagnostic accuracy [296].

### 7.2. Noise

Clinicians’ performance can be interrupted by frequent environmental distractions caused by noise, high pressure, and cognitive overload, leading to diagnostic errors and disruption of care transitions [297]. Extended exposure to noise in healthcare environments, such as voices or equipment noise, can impair cognitive function, reducing concentration, precision, and work efficiency [293,298,299]. For example, the ability to detect subtle findings during the physical exam can be reduced, particularly in noisy settings. When clinicians have difficulty hearing heart murmurs through the stethoscope, it can result in missing important information or encountering challenges in gathering it [300].

Emergency Departments (EDs), which typically have high patient volumes and urgent requests, examplify high ambient noise environments. Anxious patients and their families also make the environment more chaotic and intense. This stressful setting can impact both patients and ED staff, disturbing communication needed to assess patients’ conditions [69]. Additionally, since medical and nursing staff often need to share the same room, the cluttered and noisy space can easily disrupt their thinking and reasoning for diagnostic results [115]. Additionally, alarm fatigue desensitizes healthcare clinicians to real alerts, causing delays or missed recognition of critical patient changes and increasing diagnostic errors due to the overwhelming presence of false or non-actionable alarms [301].

### 7.3. Workstation Layout and Distance

Clinical workstations significantly influence diagnostic capabilities and patient care. Studies on the workstation’s distance and layout have shown their impact on communication and patient interaction. Clinical computer workstations should prioritize ergonomic comfort and accessibility of commonly used tools. In addition, the design of the examination table can significantly impact clinicians’ ability to collect detailed information with greater comprehensiveness and accuracy.

The workspace design and layout can facilitate communication and patient care in different ways. The shared workspace with closer proximity can enhance clinicians’ situation awareness and prompt discussion about patient care plans. For example, it is easier to see and talk with colleagues when they sit closer together, making the exchange of information about each other’s tasks and actions clearer [302]. On the other hand, the increased distance can foster more private conversations with patients and other staff members, but it may raise potential concerns regarding delayed response times and reduced peer support [303,304]. The layout of patient seat arrangements in the consultation room can also significantly influence clinician-patient interactions. For example, sitting perpendicularly represents teamwork and support, as clinicians and patients work together for one common goal. Sitting side by side may enhance teaching and communicating instructions. Sitting face-to-face can demonstrate authority and can help the doctor or clinician obtain patient compliance. If a desk or computer sits between them, it can create distance and weaken the connection [305].

Clinicians rely on the computer workstation to handle administrative tasks, billing and accounting, and sharing and editing patient medical records [306], which, as mentioned previously, can influence diagnostic processes in multiple ways. Frequently used tools, supplies, patient call lights, and phones should be within arm’s reach to reduce response time [307]. The monitor, keyboard, and mouse should remain adjustable and carefully positioned to prevent strains from prolonged use and reduce fatigue [308]. For example, a study showed that radiologists experience musculoskeletal discomfort affecting their productivity when the top of the monitor is not at eye level in a seated position or when their wrists are not straight and flat while using the mouse [309]. Although no specific study has investigated how the discomfort a clinician experiences from computer workstation setup directly affects diagnostic performance in clinical environments, a study on surgical performance showed that physical discomfort can increase cognitive load and impair motor control, which can negatively affect clinical results [310]. In addition to computing and collaboration workspaces, the design of examination workstations and tools used during the diagnostic process is important to prevent fatigue and discomfort from awkward postures during demanding tasks. Poor posture can hinder clinician concentration when assessing patients, affecting diagnostic accuracy, decision making, and overall patient care quality [311]. If assessment and procedural requirements are considered, the position of exam tables can significantly influence evaluation completeness and result accuracy [312]. For example, evaluating jugular venous pressure or inspecting the liver that needs access from the patient’s right side [313], and conventional medical practice in the United States also involves examining patients from the right side [314]. The size of the exam room and the availability of clearance space around the exam tables also determine the staff’s ability to assist with patient transfers and positioning for a more thorough examination [315]. Additionally, adjustable exam tables or lifting equipment with more flexibility can meet various patient screening needs [316].

## 8. External Environment

The external environment encompasses the broader legal, financial, and regulatory factors that impact the diagnosis process. These elements lie outside the direct control of individual patients and clinicians but significantly shape diagnostic processes and outcomes. They include patient financing and insurance systems, healthcare policies and regulations such as the U.S. Food and Drug Administration (FDA), the Office of the National Coordinator for Health Information Technology (ONC), and the Clinical Laboratory Improvement Amendments (CLIA), as well as oversight by regulatory and accrediting organizations, including licensure, certification, and accreditation systems. The external environment serves as a broad framework that interacts with and influences other SEIPS domains, providing a foundation and setting boundaries for diagnostic devices and the diagnostic process.

### 8.1. Finance & Insurance

The financing of health services includes factors such as patient income, wealth, and the capacity to afford care, as well as the actual expenses of healthcare. These expenses are shaped by insurance plans and cost-sharing obligations [317]. These financial elements can affect one’s access to timely and suitable diagnostic services, leading to potential delays or errors when affordability or insurance obstacles emerge.

Insurance-related systemic issues often create significant challenges in obtaining prompt and accurate diagnostic services, especially for low-income individuals [52]. Low reimbursement rates discourage providers from participating in insurance plans, which will reduces the availability of providers [318] and force clinicians to repeatedly negotiate with insurers for coverage of crucial procedures, further intensifying time pressures for timely diagnostic testing [319]. High out-of-pocket expenses and coverage barriers also deter patients from seeking timely medical help. Financial constraints often threaten accurate diagnosis as patients with high out-of-pocket expenses usually decline more advanced but costly imaging or tests [320]. Insurance coverage is usually based on evidence-based clinical guidelines, which are designed for the average patient and typical clinical situations. However, when a patient has comorbidities or unusual features that fall outside routine evaluation or treatment, it is often the clinician who must provide additional justification or supporting documentation to request individual consideration or to challenge unfavorable coverage decisions [321]. Additionally, many individuals with limited incomes often depend on government-sponsored health insurance programs, such as Medicaid, which are specifically designed to support at-risk populations [322]. While these programs can be helpful, they often have a very limited network of providers and complicated administrative processes. This can make it difficult for people in vulnerable situations to get the services they need, which, unfortunately, adds to existing health disparities [317].

### 8.2. Policy & Regulations—Devices and Testing

In the United States, several federal and state agencies and accreditation organizations play critical roles in regulating and developing policies for diagnostic tests/devices and hospital practices to ensure diagnostic safety, effectiveness, and quality.

The FDA plays a central role in regulating and ensuring the safety and effectiveness of various products, including drugs, biologics, and medical devices [323]. The FDA’s regulatory scope includes diagnostics that are clinician, patient, and technician-facing. Examples include in vitro diagnostic devices (IVDs), such as cholesterol panels and genetic tests, which examine human samples to support and assist in diagnosis, screening, and disease monitoring [324]. Although certain IVDs have not historically been reviewed by the FDA, they are now receiving more attention due to their broad use in diagnosing common conditions and diseases [325].

In addition to regulating the reliability and accuracy of diagnostic tools, the FDA is also responsible for overseeing the usability of diagnostic devices. The FDA guidance titled “Applying Human Factors and Usability Engineering to Medical Devices” [326] emphasizes the importance of device safety and effectiveness for the intended users, uses, and the use environments. For example, usability testing is required for diagnostic devices such as low-field magnetic resonance imaging (MRI) systems and home sleep apnea testing (HSAT) devices, as user interaction can be directly linked to outcomes. Factors such as user characteristics, interface design, and the environment of use can influence safety and effectiveness [327]. In HSAT devices, usability testing helps prevent errors such as incorrect sensor placement or failure to activate the device due to unclear displays or inadequate instructions [328].

CLIA of 1988 was enacted to ensure the accuracy, reliability, and timeliness of laboratory test results used in diagnosis, screening, and patient monitoring [329]. CLIA uses the FDA’s test complexity classification system (waived, moderate, and high complexity) to determine the level of regulatory requirements. Laboratories must comply with their specific complexity standards, which include personnel qualifications, equipment calibration, and quality assurance procedures [330]. If a laboratory violates CLIA regulations, it could face several consequences, including losing its certification, suspension of Medicare reimbursements, and a multi-year ban on operating or leading any CLIA-certified lab. The Centers for Medicare & Medicaid Services (CMS), responsible for enforcing CLIA, manages certification, inspections, proficiency testing, and compliance-related activities [331]. For example, a commercial laboratory was found to have referred proficiency testing (PT) samples between its own branches, which can artificially boost performance results and give a misleading impression of testing accuracy. Such behavior compromises the regulatory system intended to protect patient safety and ensure the reliability of tests [332].

Similarly to the FDA, the ONC ensures the usability of diagnostic products by regulating the digital infrastructure that supports them. Its policies focus on standards for EHR systems, enabling secure, seamless data exchange and retrieval essential for accurate diagnosis [333]. The ONC sponsored the creation of the Safety Assurance Factors for EHR Resilience (SAFER) guides, including checklists that help proactively identify and mitigate potential risks associated with EHR [334]. For example, practices outlined in the Patient Identification Guide for SAFER self-assessment guidelines ensures that essential patient information is prominently shown on all relevant equipment to confirm that medical services are provided to the correct patient [335]. The ONC also partners with the National Institute of Standards and Technology (NIST) and the Agency for Healthcare Research and Quality (AHRQ) to develop a comprehensive specification for an objective and repeatable procedure that measures and evaluates the usability of healthcare information technology [336].

### 8.3. Policy & Regulations—Licensure, Certification and Accreditation

Licensure, certification, and accreditation are critical for ensuring quality and safety in healthcare, as they assess and oversee healthcare organizations and clinicians based on established standards. Licensure is a mandatory process that grants individuals the legal authority to practice a profession and grants healthcare organizations the authority to operate and deliver services in compliance with regulatory standards [337]. The requirements for individuals can vary by profession or state and usually include specific examinations or educational programs [338]. For example, medical licensing examinations in the United States include various assessments across different stages of training, including assessments of diagnostic skills. These evaluations assess examinees’ ability to apply medical knowledge to patient care decisions and simulate the full diagnostic reasoning process through simulated case scenarios [339]. Some core skills and knowledge tested, such as problem-solving and clinical decision-making, have been shown to correlate with diagnostic accuracy in practice [92]. However, when faced with diagnostic errors, especially those leading to patient harm, clinicians may face malpractice claims, resulting in possible disciplinary measures from state medical boards [340], such as license suspension or revocation, probation, reprimands, fines, mandatory retraining, or practice restrictions [341]. These consequences further emphasize the importance of ongoing professional development and continuing education to help clinicians stay aligned with the evolving standards, especially for senior clinicians [342].

Certification, on the other hand, is usually voluntary [337]. It is granted by an authorized entity to assess whether an individual or organization meets specific qualifications or criteria. These usually focus on additional competencies or services that go beyond the basic requirements for licensure [343]. An example of an organization that offer certification programs focused on reducing diagnostic errors is the American Board of Internal Medicine (ABIM), which provides Maintenance of Certification (MOC) program to evaluate physicians’ diagnostic reasoning and clinical judgment through structured assessments to reinforce their decision making and appropriate use of imaging and other diagnostic tests [344,345].

Accreditation is typically a voluntary program sponsored by a non-governmental agency using external peer reviews to assess whether healthcare organizations adhere to pre-established standards [346]. Studies have found that hospital accreditation programs usually lead to improved patient safety, clinical performance, and operational efficiency, driven by stronger safety cultures, standardized procedures, and better compliance with care guidelines [347,348]. For example, the Joint Commission (TJC) is an independent, nonprofit organization that accredits hospitals in the United States to improve healthcare quality and safety through rigorous performance standards [349]. TJC-accredited hospitals often perform better on quality measures assessing the delivery of healthcare services according to evidence-based standards [350]. Accreditation plays an important role in reducing diagnostic errors. Because accreditation emphasizes improving communication and transparency, hospitals may lose accreditation or face fines if they fail to disclose diagnostic errors to patients [351]. The loss of accreditation for hospitals can lead to severe consequences, including damage to hospitals’ image and to patients’ trust [352], and loss of eligibility for federal funding and insurance reimbursement from Medicare and Medicaid, which can impact their financial stability [353,354]. Therefore, some accreditation programs provide continuing learning to further support diagnostic safety, such as the Joint Accreditation for Interprofessional Continuing Education [355]. This emphasizes lifelong learning to maintain and update clinical knowledge and promote interprofessional collaboration for effective communication, which helps reduce diagnostic errors and enhance patient safety.

## 9. Limitation

The examples and evidence referenced in this review predominantly draw on studies conducted in U.S.-based, high-income healthcare systems. As a result, some contributory factors, particularly those related to access to diagnostic tools, digital infrastructure, insurance structures, and policy and regulation, could differ substantially in low- and middle-income settings. These differences should be considered when interpreting how SEIPS-based insights may apply in other healthcare environments.

## 10. Conclusions

Using the SEIPS 3.0 model, this narrative literature review examined the various factors contributing to diagnostic errors during the patient journey. By analyzing the diagnostic work system in key areas: Diagnostic Team Members, Tasks, Technologies and Tools, Organization, Physical Environment, and External Environment, the review illustrates how each aspect and member within the diagnostic journey is interconnected and can influence the diagnostic process and its outcomes. This review also identifies several key areas for future improvement, aiming to enhance diagnostic processes and reduce errors.

## 11. Future Directions

As shown in this literature review, factors such as patients’ health literacy, communication quality, socio-behavioral factors, clinical reasoning, clinicians’ cognitive bias, tools and technology used, and organizational and administrative rules and culture play a large role in diagnostic outcomes. However, many of the contributory data are not easily extracted and are often fragmented in medical records or facility data, leaving important human and contextual information unconsidered when measuring diagnostic errors [356]. Therefore, to advance diagnostic safety, these factors should not only be recognized but also systematically quantified and measured.

To help guide stakeholders such as clinicians, researchers, and clinical administrators’ actions in improving and securing diagnostic safety, further research is needed to develop data collection tools that can capture a more comprehensive range of diagnostic factors during patient intake, clinical decision making, and care management. These tools will allow diagnostic contributory factors to be more comprehensively traced throughout the patient journey, support diagnostic error analytics at the clinical, system-wide, and national levels, and facilitate risk mitigation targeted at relevant healthcare system stakeholders (patients, clinicians, organizations, regulators, etc.). These efforts should also be extended to disease-specific diagnostic error definitions and systematic applications that align with the aforementioned NASEM definition: timing, accuracy, and communication.

Beyond identification and quantification of contributory factors and diagnostic events, downstream diagnostic safety efforts should also focus on risk mitigation of critical contributory factors. To mitigate patient risk, addressing low health literacy and improving cultural competency are both critical, as low health literacy and cultural barriers often limit patient communication and medical instruction adherence. Education approaches that are readily implemented, like health training programs, play a key role by providing basic health knowledge, such as diet, exercise, and mental health, to build fundamental disease awareness. In addition, emerging digital tools offer more convenient resources that are designed for different cultures, languages, and social groups. For example, animation [357,358], text or social media promotion [359,360,361], or apps [362,363] help patients acquire health information more effectively [364]. Therefore, patients can cultivate healthier behaviors and improve their confidence and communication skills when interacting with clinicians [365].

From the perspective of clinicians, to mitigate the influence of cognitive bias, direct integration of real scenario-based studies in future training programs, including the common types of bias, possible risks, and outcomes for patients, can help build awareness and the ability to identify cognitive bias and provide opportunities to practice slow and analytical processing [366,367]. In addition to strengthening the clinician-patient relationship, improving information transparency [368,369], such as providing easy access to medical information for patients [370], can bridge the information gap and encourage engagement.

Beyond individual training and relationship building, organizational changes are also important. Organizations can incorporate mental workload measurements into existing practices that assess task and cognitive load, rather than relying on traditional productivity metrics, such as the number of patients a clinician sees in a day or the time taken to complete a task [371,372]. This approach can better reflect realistic clinician-related outcomes, such as performance and patient care quality, and help establish standards to prevent burnout and errors [121]. Since diagnostic processes differ across care settings in terms of workflow, communication needs, and resources, organizational strategies for diagnostic safety should be tailored to each specific setting [373,374]. Inpatient and acute care settings commonly use inpatient diagnostic error triggers [375] and EHR-based interventions [376], whereas outpatient and primary care settings rely more on population risk-stratification tools [377] and ambulatory e-triggers targeting missed follow-up or unexpected ED visits [378]. Future work should account for these contextual differences while also considering cross-setting integration to support diagnostic safety as patients transition across care environments. Finally, to foster a culture of continuous learning and accountability, creating a non-punitive error reporting system allows all medical staff to be heard and appreciated by leadership, which encourages the healthcare team to learn from mistakes and share their professional insights [379].

## Figures and Tables

**Table 1:** Search Terms for Literature Identification.

SEIPS Categories	Search Terms
Diagnostic Team Member	logistic barrier, medical resource imbalance, telemedicine infrastructure in rural area, referral delay, health literacy, symptom recognition, patient–clinician communication, patient mental health, cultural and social disparities, social stigma, clinician knowledge, clinician expertise, clinician cognitive bias, heuristic, anchoring bias, attribution bias, availability bias, confidence bias, delay discounting
Tasks	diagnostic process, symptom presentation, overlapping symptoms, clinical guidelines, clinical decision-making tasks
Technologies and Tools	EHR data, EHR usability, HER documentation quality, diagnostic test accuracy, diagnostic test accessibility, clinical decision support, clinical checklist, clinical decision support system, remote communication
Organization	clinical organizational culture, clinical power hierarchy, peer pressure, diagnostic repercussion, malpractice consequence, handoffs, clinical team coordination, clinical team communication, interdisciplinary collaboration, appointment scheduling, limited available schedule, consultation time constraint, lab testing turnaround, equipment availability
Physical Environment	lighting, noise, ergonomics, workspace design, environmental distractions
External Environment	insurance policy, coverage limitations, regulatory requirements, FDA medical device usability, licensure and certification requirements, accreditation standards

## Data Availability

No new data were created or analyzed in this study. Data sharing is not applicable to this article.
